# Supplementary material for: Identification of a super-functional Tfh-like subpopulation in murine lupus by pattern perception
Source: eLife. 2020 May 22;9:e53226. doi: 10.7554/eLife.53226 (PMC7274784; doi:10.7554/eLife.53226)
Supplement: Source code 1. [file elife-53226-code1.zip › source code 1-PRI_code.html]

Data analysis with PRI - Pattern Recognition with Immune cells


Code 

- Show All Code
- Hide All Code
- Download Rmd

# Data analysis with PRI - Pattern Recognition with Immune cells

#### *Yen Hoang*

#### *22 July 2019*


## Data description

The female filial 1 generation of NZBxW mice was used for this study. Tissue from spleen, kidney, liver and lung cells were extracted, prepared and stained according to the guidelines for the use of flow cytometry and cell sorting in immunological studies. The data set can be downloaded from FlowRepository. Compensation, elimination of dublets and dead cells, gating to CD44+ T cells were pre-processed with simple two dimensional scatter plots in FlowJo.

## Example analysis with PRI - Pattern Recognition with Immune cells

We chose two examples how to create the binplots with three and four marker combinations, respectively. Only the parameters in the beginning need to be adjusted.

### Load libraries

We operated on R v3.2.4 Revised. Library ‘flowCore’ v1.36.9 was installed via BioConductor to read the fcs-files. Help and plot functions are accumulated in ‘YH\_bintriploT\_functions.R’.


```
rm(list = ls())
fcs = new.env()
library(flowCore)
source("/data/PRI manuscript/R/standalone/YH_binplot_functions.R")
```

### Data import

The file “TL160721\_OHiPU\_S23\_CD4.fcs” was used to create Figure 1e+f and has 334276 cells after preprocession. The file “export\_SG20170627\_4hP+I\_011\_CD44.fcs” was used to create Figure 6e and has 73639 cells after preprocession. Cells were transformed with inverse hyperbolic sine (asinh), which is a common transformation method for flow cytometry data [doi:10.1186/1471-2105-11-546]. Change the path according to your download folder.


```
path.folder = file.path("","data","PRI\ manuscript","R","fcs_examples")
file.tri = "TL160721_OHiPU_S23_CD4.fcs"
file.quad = "export_SG20170627_4hP+I_011_CD44.fcs"
####### data for bin plots with 3 parameters (example from Figure 1e+f)
# read the alias vs channel mapping from keyword() option
data.tri = read.FCS(file=file.path(path.folder,file.tri),which.lines=1)
keys.tri = keyword(data.tri)
aliases = unlist(keyword(data.tri, c("$P1N","$P2N","$P3N","$P4N","$P5N",
                                     "$P6S","$P7S","$P8S","$P9S","$P10S",
                                     "$P11S","$P12S","$P13S","$P14S","$P15S",
                                     "$P16S")))
channels = unlist(keyword(data.tri,c("$P1N","$P2N","$P3N","$P4N","$P5N",
                                     "$P6N","$P7N","$P8N","$P9N","$P10N",
                                     "$P11N","$P12N","$P13N","$P14N","$P15N",
                                     "$P16N")))
map.tri <- data.frame(alias = aliases, channels = channels)
# load data
exprs.tri= as.data.frame(exprs(read.FCS(file=file.path(path.folder,file.tri),
                    channel_alias = map.tri, transformation=NULL)))
# rename column names
for ( i in 1:ncol(exprs.tri)) {
  colnames(exprs.tri)[i] = strsplit(colnames(exprs.tri)[i]," ")[[1]][1]
}
# transform data with asinh()
exprs.tri= asinh(exprs.tri)
########################################
####### data for bin plots with 4 parameters (example from Figure 6e)
data.quad = read.FCS(file=file.path(path.folder,file.quad),which.lines=1)
keys.quad = keyword(data.quad)
aliases.quad = unlist(keyword(data.quad,c("$P1N","$P2N","$P3N","$P4N","$P5N",
                                     "$P6S","$P7S","$P8S","$P9S","$P10S",
                                     "$P11S","$P12S","$P13S")))
channels.quad = unlist(keyword(data.quad,c("$P1N","$P2N","$P3N","$P4N","$P5N",
                                     "$P6N","$P7N","$P8N","$P9N","$P10N",
                                     "$P11N","$P12N","$P13N")))
map.quad <- data.frame(alias = aliases.quad, channels = channels.quad)
# load data
exprs.quad= as.data.frame(exprs(read.FCS(file=file.path(path.folder,file.quad),
                    channel_alias = map.quad, transformation=NULL)))
# rename column names
for ( i in 1:ncol(exprs.quad)) {
  colnames(exprs.quad)[i] = strsplit(colnames(exprs.quad)[i]," ")[[1]][1]
}
colnames(exprs.quad)[5] = "IFN-g"
colnames(exprs.quad)[10] = "CXCR5"
# transform data with asinh()
exprs.quad= asinh(exprs.quad)
########################################
```

### binplot with three parameters

The range of parameter X (TNF-a) and parameter Y (CD44) on x and y axis, respectively, are categorized into bins of size 0.2x0.2. With the cells in each bin, different statistical methods can be plotted into a color-coded manner. Here, cell density and mean fluorescence intensity of parameter Z (MFI) are displayed in pseudo-color. All parameters were included in the analysis and bins with less than 10 cells were excluded. This minimum count of cells was used to balance the impact of identifying comparatively rare subpopulations while retaining the statistical power. Examples are shown from Figure 1e.


```
### INITIATING PARAMETERS
featX = "TNFa"
featY = "CD44"
featZ1 = "IFNg"
cutoffs.man = c(6.6,9,5)
binSize = 0.2
### PLOTTING PARAMETERS FOR TWO PLOTS
par(mfrow = c(1,2),
    cex.lab = 1.4, cex.axis = 1.4,
    mgp = c(2.0, 0, 0),
    mar = c(2.4,2,2.5,0),
    oma = c(1,1,1,1))
### CALL PLOT FUNCTIONS
fcs$binplot_table(
  data = exprs.tri, 
  feat.X = featX, 
  feat.Y = featY, 
  feat.Z1 = featZ1, 
  calc = "density",
  binsize = binSize,
  cutoffs = cutoffs.man
)
fcs$binplot_table(
  data = exprs.tri, 
  feat.X = featX, 
  feat.Y = featY, 
  feat.Z1 = featZ1, 
  calc = "MFI",
  binsize = binSize,
  cutoffs = cutoffs.man
)
```

### Binplot with three parameters and using cutoff of parameter Z

The use of the cutoff of parameter Z can result in an additional advantage. Frequency of parameter Z (IFN-g) producing cells (%) and mean fluorescence intensity of parameter Z producing cells (MFI+) can then be displayed. Examples are shown from Figure 1e+1f.


```
### INITIATING PARAMETERS
featX = "TNFa"
featY = "CD44"
featZ1 = "IFNg"
cutoffs.man = c(6.6,9,5)
binSize = 0.2
### PLOTTING PARAMETERS FOR TWO PLOTS
par(mfrow = c(1,2),
    cex.lab = 1.4, cex.axis = 1.4,
    mgp = c(2.0, 0, 0),
    mar = c(2.4,2,2.5,0),
    oma = c(1,1,1,1))
### CALL PLOT FUNCTIONS
  
fcs$binplot_table(
  data = exprs.tri, 
  feat.X = featX, 
  feat.Y = featY, 
  feat.Z1 = featZ1, 
  calc = "freq",
  binsize = binSize,
  cutoffs = cutoffs.man
)
fcs$binplot_table(
  data = exprs.tri, 
  feat.X = featX, 
  feat.Y = featY, 
  feat.Z1 = featZ1, 
  calc = "MFI+",
  binsize = binSize,
  cutoffs = cutoffs.man
)
```

### Binplot with four parameters

Additionally, frequency of double producing cells (parameter Z1 and Z2) are introduced in green scales. Examples are shown from Figure 6e.


```
### INITIATING PARAMETERS
featX = "PD1"
featY = "IFNg"
featZ1 = "CXCR5"
featZ2 = "Bcl6"
cutoffs.man = c(6.982,7.479,7.783,7.346)
binSize = 0.2
### plotting options
maxfreq = 80
### PLOTTING PARAMETERS FOR TWO PLOTS
par(mfrow = c(1,2),
    cex.lab = 1.4, cex.axis = 1.4,
    mgp = c(2.0, 0, 0),
    mar = c(2,2,2,0),
    oma = c(2,2,2,1))
### PLOT BIN CONSTRUCT
fcs$binplot_construct(
  data = exprs.quad,
  feat.X = featX,
  feat.Y = featY,
  binsize = binSize,
  plot.range = c(3,11,3,11)
)
### PLOT HEATMAP
fcs$binplot_freq_doublepos(
  data = exprs.quad,
  feat.X = featX,
  feat.Y = featY,
  feat.Z1 = featZ1,
  feat.Z2 = featZ2,
  cutoffs = cutoffs.man,
  maxfreq = maxfreq,
  binsize = binSize,
  plot.range = c(3,11,3,11)
)
```


```
############## PLOT #2
### INITIATING PARAMETERS
featX = "PD1"
featY = "IFNg"
featZ1 = "IL21"
featZ2 = "Bcl6"
cutoffs.man = c(6.982,7.479,7.696,7.346)
binSize = 0.2
### plotting options
maxfreq = 50
### PLOT BIN CONSTRUCT
fcs$binplot_construct(
  data = exprs.quad,
  feat.X = featX,
  feat.Y = featY,
  binsize = binSize,
  plot.range = c(3,11,3,11)
)
### PLOT HEATMAP
fcs$binplot_freq_doublepos(
  data = exprs.quad,
  feat.X = featX,
  feat.Y = featY,
  feat.Z1 = featZ1,
  feat.Z2 = featZ2,
  cutoffs = cutoffs.man,
  binsize = binSize,
  maxfreq = maxfreq,
  plot.range = c(3,11,3,11)
)
```


In regards to 3D-surface plots, frequency bin tables are provided and exported from PRI and created with package ‘plotly’ v4.7.1. Examples are shown from Figure 6g.


```
library(plotly)
featX = "PD1"
featY = "IFNg"
featZ1 = "Bcl6"
cutoffs.man = c(6.982,7.479,7.346)
binSize = 0.2
binplot.table.Z1 = fcs$binplot_table(
  data = exprs.quad, 
  feat.X = featX, 
  feat.Y = featY, 
  feat.Z1 = featZ1, 
  calc = "freq",
  binsize = binSize,
  cutoffs = cutoffs.man,
  plotting = FALSE
)
featZ2 = "IL21"
cutoffs.man = c(6.982,7.479,7.696)
binplot.table.Z2 = fcs$binplot_table(
  data = exprs.quad, 
  feat.X = featX, 
  feat.Y = featY, 
  feat.Z1 = featZ2, 
  calc = "freq",
  binsize = binSize,
  cutoffs = cutoffs.man,
  plotting = FALSE
)
x=seq(2,12,by=0.2)
y=seq(2,12,by=0.2)
scene = list(xaxis=list(title= "PD-1"),
    yaxis=list(title="IFN-g"),
    zaxis=list(title="freq",range=c(0,85)),
    cameraposition=list(c(-0.1, 0.4, -1, 0.33), c(0.0, 0, 0.0), 2)
)
### UNCOMMENT TO PLOT
### Figure with Bcl6
# plot_ly(showscale = TRUE) %>% add_surface(x=x,y=y,z=t(binplot.table.Z1), cmin=0, cmax=82,colors="Greens",reversescale=F) %>% layout(scene=scene)
### Figure with IL21 
# plot_ly(showscale = TRUE) %>% add_surface(x=x,y=y,z=t(binplot.table.Z2), cmin=0, cmax=82,colors="Greens",reversescale=F) %>% layout(scene=scene)
```

LS0tCnRpdGxlOiAiRGF0YSBhbmFseXNpcyB3aXRoIFBSSSAtIFBhdHRlcm4gUmVjb2duaXRpb24gd2l0aCBJbW11bmUgY2VsbHMiCmF1dGhvcjogIlllbiBIb2FuZyIKZGF0ZTogIjIyIEp1bHkgMjAxOSIKb3V0cHV0OiBodG1sX25vdGVib29rCi0tLQoKYGBge3Igc2V0dXAsIGluY2x1ZGU9RkFMU0V9CmtuaXRyOjpvcHRzX2NodW5rJHNldChlY2hvID0gVFJVRSkKYGBgCgoKIyMgRGF0YSBkZXNjcmlwdGlvbgoKVGhlIGZlbWFsZSBmaWxpYWwgMSBnZW5lcmF0aW9uIG9mIE5aQnhXIG1pY2Ugd2FzIHVzZWQgZm9yIHRoaXMgc3R1ZHkuIFRpc3N1ZSBmcm9tIHNwbGVlbiwga2lkbmV5LCBsaXZlciBhbmQgbHVuZyBjZWxscyB3ZXJlIGV4dHJhY3RlZCwgcHJlcGFyZWQgYW5kIHN0YWluZWQgYWNjb3JkaW5nIHRvIHRoZSBndWlkZWxpbmVzIGZvciB0aGUgdXNlIG9mIGZsb3cgY3l0b21ldHJ5IGFuZCBjZWxsIHNvcnRpbmcgaW4gaW1tdW5vbG9naWNhbCBzdHVkaWVzLiBUaGUgZGF0YSBzZXQgY2FuIGJlIGRvd25sb2FkZWQgZnJvbSBbRmxvd1JlcG9zaXRvcnldKGh0dHA6Ly9mbG93cmVwb3NpdG9yeS5vcmcpLiBDb21wZW5zYXRpb24sIGVsaW1pbmF0aW9uIG9mIGR1YmxldHMgYW5kIGRlYWQgY2VsbHMsIGdhdGluZyB0byBDRDQ0KyBUIGNlbGxzIHdlcmUgcHJlLXByb2Nlc3NlZCB3aXRoIHNpbXBsZSB0d28gZGltZW5zaW9uYWwgc2NhdHRlciBwbG90cyBpbiAgW0Zsb3dKb10oaHR0cDovL2Zsb3dqby5jb20pLgoKIyMgRXhhbXBsZSBhbmFseXNpcyB3aXRoIFBSSSAtIFBhdHRlcm4gUmVjb2duaXRpb24gd2l0aCBJbW11bmUgY2VsbHMKV2UgY2hvc2UgdHdvIGV4YW1wbGVzIGhvdyB0byBjcmVhdGUgdGhlIGJpbnBsb3RzIHdpdGggdGhyZWUgYW5kIGZvdXIgbWFya2VyIGNvbWJpbmF0aW9ucywgcmVzcGVjdGl2ZWx5LiBPbmx5IHRoZSBwYXJhbWV0ZXJzIGluIHRoZSBiZWdpbm5pbmcgbmVlZCB0byBiZSBhZGp1c3RlZC4KCiMjIyBMb2FkIGxpYnJhcmllcwpXZSBvcGVyYXRlZCBvbiBbUiB2My4yLjQgUmV2aXNlZF0oaHR0cDovL3d3dy5yLXByb2plY3Qub3JnKS4gTGlicmFyeSAnZmxvd0NvcmUnIHYxLjM2Ljkgd2FzIGluc3RhbGxlZCB2aWEgW0Jpb0NvbmR1Y3Rvcl0oaHR0cHM6Ly93d3cuYmlvY29uZHVjdG9yLm9yZy8pIHRvIHJlYWQgdGhlIGZjcy1maWxlcy4gSGVscCBhbmQgcGxvdCBmdW5jdGlvbnMgYXJlIGFjY3VtdWxhdGVkIGluICdZSF9iaW50cmlwbG9UX2Z1bmN0aW9ucy5SJy4gCmBgYHtyIGxvYWQgZnVuY3Rpb25zfQpybShsaXN0ID0gbHMoKSkKZmNzID0gbmV3LmVudigpCmxpYnJhcnkoZmxvd0NvcmUpCnNvdXJjZSgiL2RhdGEvUFJJIG1hbnVzY3JpcHQvUi9zdGFuZGFsb25lL1lIX2JpbnBsb3RfZnVuY3Rpb25zLlIiKQpgYGAKCgojIyMgRGF0YSBpbXBvcnQKVGhlIGZpbGUgIlRMMTYwNzIxX09IaVBVX1MyM19DRDQuZmNzIiB3YXMgdXNlZCB0byBjcmVhdGUgRmlndXJlIDFlK2YgYW5kIGhhcyAzMzQyNzYgY2VsbHMgYWZ0ZXIgcHJlcHJvY2Vzc2lvbi4gVGhlIGZpbGUgImV4cG9ydF9TRzIwMTcwNjI3XzRoUCtJXzAxMV9DRDQ0LmZjcyIgd2FzIHVzZWQgdG8gY3JlYXRlIEZpZ3VyZSA2ZSBhbmQgaGFzIDczNjM5IGNlbGxzIGFmdGVyIHByZXByb2Nlc3Npb24uIENlbGxzIHdlcmUgdHJhbnNmb3JtZWQgd2l0aCBpbnZlcnNlIGh5cGVyYm9saWMgc2luZSAoYXNpbmgpLCB3aGljaCBpcyBhIGNvbW1vbiB0cmFuc2Zvcm1hdGlvbiBtZXRob2QgZm9yIGZsb3cgY3l0b21ldHJ5IGRhdGEgW2RvaToxMC4xMTg2LzE0NzEtMjEwNS0xMS01NDZdLiBDaGFuZ2UgdGhlIHBhdGggYWNjb3JkaW5nIHRvIHlvdXIgZG93bmxvYWQgZm9sZGVyLiAKYGBge3IgZGF0YSBpbXBvcnQxfQpwYXRoLmZvbGRlciA9IGZpbGUucGF0aCgiIiwiZGF0YSIsIlBSSVwgbWFudXNjcmlwdCIsIlIiLCJmY3NfZXhhbXBsZXMiKQoKZmlsZS50cmkgPSAiVEwxNjA3MjFfT0hpUFVfUzIzX0NENC5mY3MiCmZpbGUucXVhZCA9ICJleHBvcnRfU0cyMDE3MDYyN180aFArSV8wMTFfQ0Q0NC5mY3MiCgojIyMjIyMjIGRhdGEgZm9yIGJpbiBwbG90cyB3aXRoIDMgcGFyYW1ldGVycyAoZXhhbXBsZSBmcm9tIEZpZ3VyZSAxZStmKQojIHJlYWQgdGhlIGFsaWFzIHZzIGNoYW5uZWwgbWFwcGluZyBmcm9tIGtleXdvcmQoKSBvcHRpb24KZGF0YS50cmkgPSByZWFkLkZDUyhmaWxlPWZpbGUucGF0aChwYXRoLmZvbGRlcixmaWxlLnRyaSksd2hpY2gubGluZXM9MSkKa2V5cy50cmkgPSBrZXl3b3JkKGRhdGEudHJpKQphbGlhc2VzID0gdW5saXN0KGtleXdvcmQoZGF0YS50cmksIGMoIiRQMU4iLCIkUDJOIiwiJFAzTiIsIiRQNE4iLCIkUDVOIiwKICAgICAgICAgICAgICAgICAgICAgICAgICAgICAgICAgICAgICIkUDZTIiwiJFA3UyIsIiRQOFMiLCIkUDlTIiwiJFAxMFMiLAogICAgICAgICAgICAgICAgICAgICAgICAgICAgICAgICAgICAgIiRQMTFTIiwiJFAxMlMiLCIkUDEzUyIsIiRQMTRTIiwiJFAxNVMiLAogICAgICAgICAgICAgICAgICAgICAgICAgICAgICAgICAgICAgIiRQMTZTIikpKQpjaGFubmVscyA9IHVubGlzdChrZXl3b3JkKGRhdGEudHJpLGMoIiRQMU4iLCIkUDJOIiwiJFAzTiIsIiRQNE4iLCIkUDVOIiwKICAgICAgICAgICAgICAgICAgICAgICAgICAgICAgICAgICAgICIkUDZOIiwiJFA3TiIsIiRQOE4iLCIkUDlOIiwiJFAxME4iLAogICAgICAgICAgICAgICAgICAgICAgICAgICAgICAgICAgICAgIiRQMTFOIiwiJFAxMk4iLCIkUDEzTiIsIiRQMTROIiwiJFAxNU4iLAogICAgICAgICAgICAgICAgICAgICAgICAgICAgICAgICAgICAgIiRQMTZOIikpKQptYXAudHJpIDwtIGRhdGEuZnJhbWUoYWxpYXMgPSBhbGlhc2VzLCBjaGFubmVscyA9IGNoYW5uZWxzKQoKIyBsb2FkIGRhdGEKZXhwcnMudHJpPSBhcy5kYXRhLmZyYW1lKGV4cHJzKHJlYWQuRkNTKGZpbGU9ZmlsZS5wYXRoKHBhdGguZm9sZGVyLGZpbGUudHJpKSwKICAgICAgICAgICAgICAgICAgICBjaGFubmVsX2FsaWFzID0gbWFwLnRyaSwgdHJhbnNmb3JtYXRpb249TlVMTCkpKQojIHJlbmFtZSBjb2x1bW4gbmFtZXMKZm9yICggaSBpbiAxOm5jb2woZXhwcnMudHJpKSkgewogIGNvbG5hbWVzKGV4cHJzLnRyaSlbaV0gPSBzdHJzcGxpdChjb2xuYW1lcyhleHBycy50cmkpW2ldLCIgIilbWzFdXVsxXQp9CiMgdHJhbnNmb3JtIGRhdGEgd2l0aCBhc2luaCgpCmV4cHJzLnRyaT0gYXNpbmgoZXhwcnMudHJpKQojIyMjIyMjIyMjIyMjIyMjIyMjIyMjIyMjIyMjIyMjIyMjIyMjIyMjCgojIyMjIyMjIGRhdGEgZm9yIGJpbiBwbG90cyB3aXRoIDQgcGFyYW1ldGVycyAoZXhhbXBsZSBmcm9tIEZpZ3VyZSA2ZSkKZGF0YS5xdWFkID0gcmVhZC5GQ1MoZmlsZT1maWxlLnBhdGgocGF0aC5mb2xkZXIsZmlsZS5xdWFkKSx3aGljaC5saW5lcz0xKQprZXlzLnF1YWQgPSBrZXl3b3JkKGRhdGEucXVhZCkKYWxpYXNlcy5xdWFkID0gdW5saXN0KGtleXdvcmQoZGF0YS5xdWFkLGMoIiRQMU4iLCIkUDJOIiwiJFAzTiIsIiRQNE4iLCIkUDVOIiwKICAgICAgICAgICAgICAgICAgICAgICAgICAgICAgICAgICAgICIkUDZTIiwiJFA3UyIsIiRQOFMiLCIkUDlTIiwiJFAxMFMiLAogICAgICAgICAgICAgICAgICAgICAgICAgICAgICAgICAgICAgIiRQMTFTIiwiJFAxMlMiLCIkUDEzUyIpKSkKY2hhbm5lbHMucXVhZCA9IHVubGlzdChrZXl3b3JkKGRhdGEucXVhZCxjKCIkUDFOIiwiJFAyTiIsIiRQM04iLCIkUDROIiwiJFA1TiIsCiAgICAgICAgICAgICAgICAgICAgICAgICAgICAgICAgICAgICAiJFA2TiIsIiRQN04iLCIkUDhOIiwiJFA5TiIsIiRQMTBOIiwKICAgICAgICAgICAgICAgICAgICAgICAgICAgICAgICAgICAgICIkUDExTiIsIiRQMTJOIiwiJFAxM04iKSkpCm1hcC5xdWFkIDwtIGRhdGEuZnJhbWUoYWxpYXMgPSBhbGlhc2VzLnF1YWQsIGNoYW5uZWxzID0gY2hhbm5lbHMucXVhZCkKCiMgbG9hZCBkYXRhCmV4cHJzLnF1YWQ9IGFzLmRhdGEuZnJhbWUoZXhwcnMocmVhZC5GQ1MoZmlsZT1maWxlLnBhdGgocGF0aC5mb2xkZXIsZmlsZS5xdWFkKSwKICAgICAgICAgICAgICAgICAgICBjaGFubmVsX2FsaWFzID0gbWFwLnF1YWQsIHRyYW5zZm9ybWF0aW9uPU5VTEwpKSkKIyByZW5hbWUgY29sdW1uIG5hbWVzCmZvciAoIGkgaW4gMTpuY29sKGV4cHJzLnF1YWQpKSB7CiAgY29sbmFtZXMoZXhwcnMucXVhZClbaV0gPSBzdHJzcGxpdChjb2xuYW1lcyhleHBycy5xdWFkKVtpXSwiICIpW1sxXV1bMV0KfQpjb2xuYW1lcyhleHBycy5xdWFkKVs1XSA9ICJJRk4tZyIKY29sbmFtZXMoZXhwcnMucXVhZClbMTBdID0gIkNYQ1I1IgojIHRyYW5zZm9ybSBkYXRhIHdpdGggYXNpbmgoKQpleHBycy5xdWFkPSBhc2luaChleHBycy5xdWFkKQoKIyMjIyMjIyMjIyMjIyMjIyMjIyMjIyMjIyMjIyMjIyMjIyMjIyMjIwpgYGAKCiMjIyBiaW5wbG90IHdpdGggdGhyZWUgcGFyYW1ldGVycwpUaGUgcmFuZ2Ugb2YgcGFyYW1ldGVyIFggKFRORi1hKSBhbmQgcGFyYW1ldGVyIFkgKENENDQpIG9uIHggYW5kIHkgYXhpcywgcmVzcGVjdGl2ZWx5LCBhcmUgY2F0ZWdvcml6ZWQgaW50byBiaW5zIG9mIHNpemUgMC4yeDAuMi4gV2l0aCB0aGUgY2VsbHMgaW4gZWFjaCBiaW4sIGRpZmZlcmVudCBzdGF0aXN0aWNhbCBtZXRob2RzIGNhbiBiZSBwbG90dGVkIGludG8gYSBjb2xvci1jb2RlZCBtYW5uZXIuIEhlcmUsIGNlbGwgZGVuc2l0eSBhbmQgbWVhbiBmbHVvcmVzY2VuY2UgaW50ZW5zaXR5IG9mIHBhcmFtZXRlciBaIChNRkkpIGFyZSBkaXNwbGF5ZWQgaW4gcHNldWRvLWNvbG9yLiBBbGwgcGFyYW1ldGVycyB3ZXJlIGluY2x1ZGVkIGluIHRoZSBhbmFseXNpcyBhbmQgYmlucyB3aXRoIGxlc3MgdGhhbiAxMCBjZWxscyB3ZXJlIGV4Y2x1ZGVkLiBUaGlzIG1pbmltdW0gY291bnQgb2YgY2VsbHMgd2FzIHVzZWQgdG8gYmFsYW5jZSB0aGUgaW1wYWN0IG9mIGlkZW50aWZ5aW5nIGNvbXBhcmF0aXZlbHkgcmFyZSBzdWJwb3B1bGF0aW9ucyB3aGlsZSByZXRhaW5pbmcgdGhlIHN0YXRpc3RpY2FsIHBvd2VyLiBFeGFtcGxlcyBhcmUgc2hvd24gZnJvbSBGaWd1cmUgMWUuCgpgYGB7ciB0cmlwbG90MX0KIyMjIElOSVRJQVRJTkcgUEFSQU1FVEVSUwpmZWF0WCA9ICJUTkZhIgpmZWF0WSA9ICJDRDQ0IgpmZWF0WjEgPSAiSUZOZyIKY3V0b2Zmcy5tYW4gPSBjKDYuNiw5LDUpCmJpblNpemUgPSAwLjIKCiMjIyBQTE9UVElORyBQQVJBTUVURVJTIEZPUiBUV08gUExPVFMKcGFyKG1mcm93ID0gYygxLDIpLAogICAgY2V4LmxhYiA9IDEuNCwgY2V4LmF4aXMgPSAxLjQsCiAgICBtZ3AgPSBjKDIuMCwgMCwgMCksCiAgICBtYXIgPSBjKDIuNCwyLDIuNSwwKSwKICAgIG9tYSA9IGMoMSwxLDEsMSkpCgojIyMgQ0FMTCBQTE9UIEZVTkNUSU9OUwpmY3MkYmlucGxvdF90YWJsZSgKICBkYXRhID0gZXhwcnMudHJpLCAKICBmZWF0LlggPSBmZWF0WCwgCiAgZmVhdC5ZID0gZmVhdFksIAogIGZlYXQuWjEgPSBmZWF0WjEsIAogIGNhbGMgPSAiZGVuc2l0eSIsCiAgYmluc2l6ZSA9IGJpblNpemUsCiAgY3V0b2ZmcyA9IGN1dG9mZnMubWFuCikKCmZjcyRiaW5wbG90X3RhYmxlKAogIGRhdGEgPSBleHBycy50cmksIAogIGZlYXQuWCA9IGZlYXRYLCAKICBmZWF0LlkgPSBmZWF0WSwgCiAgZmVhdC5aMSA9IGZlYXRaMSwgCiAgY2FsYyA9ICJNRkkiLAogIGJpbnNpemUgPSBiaW5TaXplLAogIGN1dG9mZnMgPSBjdXRvZmZzLm1hbgopCmBgYAoKIyMjIEJpbnBsb3Qgd2l0aCB0aHJlZSBwYXJhbWV0ZXJzIGFuZCB1c2luZyBjdXRvZmYgb2YgcGFyYW1ldGVyIFoKVGhlIHVzZSBvZiB0aGUgY3V0b2ZmIG9mIHBhcmFtZXRlciBaIGNhbiByZXN1bHQgaW4gYW4gYWRkaXRpb25hbCBhZHZhbnRhZ2UuIEZyZXF1ZW5jeSBvZiBwYXJhbWV0ZXIgWiAoSUZOLWcpIHByb2R1Y2luZyBjZWxscyAoJSkgYW5kIG1lYW4gZmx1b3Jlc2NlbmNlIGludGVuc2l0eSBvZiBwYXJhbWV0ZXIgWiBwcm9kdWNpbmcgY2VsbHMgKE1GSSspIGNhbiB0aGVuIGJlIGRpc3BsYXllZC4gIEV4YW1wbGVzIGFyZSBzaG93biBmcm9tIEZpZ3VyZSAxZSsxZi4KYGBge3IgdHJpcGxvdDJ9CiMjIyBJTklUSUFUSU5HIFBBUkFNRVRFUlMKZmVhdFggPSAiVE5GYSIKZmVhdFkgPSAiQ0Q0NCIKZmVhdFoxID0gIklGTmciCmN1dG9mZnMubWFuID0gYyg2LjYsOSw1KQpiaW5TaXplID0gMC4yCgojIyMgUExPVFRJTkcgUEFSQU1FVEVSUyBGT1IgVFdPIFBMT1RTCnBhcihtZnJvdyA9IGMoMSwyKSwKICAgIGNleC5sYWIgPSAxLjQsIGNleC5heGlzID0gMS40LAogICAgbWdwID0gYygyLjAsIDAsIDApLAogICAgbWFyID0gYygyLjQsMiwyLjUsMCksCiAgICBvbWEgPSBjKDEsMSwxLDEpKQojIyMgQ0FMTCBQTE9UIEZVTkNUSU9OUwogIApmY3MkYmlucGxvdF90YWJsZSgKICBkYXRhID0gZXhwcnMudHJpLCAKICBmZWF0LlggPSBmZWF0WCwgCiAgZmVhdC5ZID0gZmVhdFksIAogIGZlYXQuWjEgPSBmZWF0WjEsIAogIGNhbGMgPSAiZnJlcSIsCiAgYmluc2l6ZSA9IGJpblNpemUsCiAgY3V0b2ZmcyA9IGN1dG9mZnMubWFuCikKCmZjcyRiaW5wbG90X3RhYmxlKAogIGRhdGEgPSBleHBycy50cmksIAogIGZlYXQuWCA9IGZlYXRYLCAKICBmZWF0LlkgPSBmZWF0WSwgCiAgZmVhdC5aMSA9IGZlYXRaMSwgCiAgY2FsYyA9ICJNRkkrIiwKICBiaW5zaXplID0gYmluU2l6ZSwKICBjdXRvZmZzID0gY3V0b2Zmcy5tYW4KKQpgYGAKCgoKIyMjIEJpbnBsb3Qgd2l0aCBmb3VyIHBhcmFtZXRlcnMKQWRkaXRpb25hbGx5LCBmcmVxdWVuY3kgb2YgZG91YmxlIHByb2R1Y2luZyBjZWxscyAocGFyYW1ldGVyIFoxIGFuZCBaMikgYXJlIGludHJvZHVjZWQgaW4gZ3JlZW4gc2NhbGVzLiBFeGFtcGxlcyBhcmUgc2hvd24gZnJvbSBGaWd1cmUgNmUuCmBgYHtyIHF1YWRwbG90fQojIyMgSU5JVElBVElORyBQQVJBTUVURVJTCmZlYXRYID0gIlBEMSIKZmVhdFkgPSAiSUZOZyIKZmVhdFoxID0gIkNYQ1I1IgpmZWF0WjIgPSAiQmNsNiIKY3V0b2Zmcy5tYW4gPSBjKDYuOTgyLDcuNDc5LDcuNzgzLDcuMzQ2KQpiaW5TaXplID0gMC4yCiMjIyBwbG90dGluZyBvcHRpb25zCm1heGZyZXEgPSA4MAoKIyMjIFBMT1RUSU5HIFBBUkFNRVRFUlMgRk9SIFRXTyBQTE9UUwpwYXIobWZyb3cgPSBjKDEsMiksCiAgICBjZXgubGFiID0gMS40LCBjZXguYXhpcyA9IDEuNCwKICAgIG1ncCA9IGMoMi4wLCAwLCAwKSwKICAgIG1hciA9IGMoMiwyLDIsMCksCiAgICBvbWEgPSBjKDIsMiwyLDEpKQoKIyMjIFBMT1QgQklOIENPTlNUUlVDVApmY3MkYmlucGxvdF9jb25zdHJ1Y3QoCiAgZGF0YSA9IGV4cHJzLnF1YWQsCiAgZmVhdC5YID0gZmVhdFgsCiAgZmVhdC5ZID0gZmVhdFksCiAgYmluc2l6ZSA9IGJpblNpemUsCiAgcGxvdC5yYW5nZSA9IGMoMywxMSwzLDExKQopCgojIyMgUExPVCBIRUFUTUFQCmZjcyRiaW5wbG90X2ZyZXFfZG91YmxlcG9zKAogIGRhdGEgPSBleHBycy5xdWFkLAogIGZlYXQuWCA9IGZlYXRYLAogIGZlYXQuWSA9IGZlYXRZLAogIGZlYXQuWjEgPSBmZWF0WjEsCiAgZmVhdC5aMiA9IGZlYXRaMiwKICBjdXRvZmZzID0gY3V0b2Zmcy5tYW4sCiAgbWF4ZnJlcSA9IG1heGZyZXEsCiAgYmluc2l6ZSA9IGJpblNpemUsCiAgcGxvdC5yYW5nZSA9IGMoMywxMSwzLDExKQopCgojIyMjIyMjIyMjIyMjIyBQTE9UICMyCiMjIyBJTklUSUFUSU5HIFBBUkFNRVRFUlMKZmVhdFggPSAiUEQxIgpmZWF0WSA9ICJJRk5nIgpmZWF0WjEgPSAiSUwyMSIKZmVhdFoyID0gIkJjbDYiCmN1dG9mZnMubWFuID0gYyg2Ljk4Miw3LjQ3OSw3LjY5Niw3LjM0NikKYmluU2l6ZSA9IDAuMgojIyMgcGxvdHRpbmcgb3B0aW9ucwptYXhmcmVxID0gNTAKCgojIyMgUExPVCBCSU4gQ09OU1RSVUNUCmZjcyRiaW5wbG90X2NvbnN0cnVjdCgKICBkYXRhID0gZXhwcnMucXVhZCwKICBmZWF0LlggPSBmZWF0WCwKICBmZWF0LlkgPSBmZWF0WSwKICBiaW5zaXplID0gYmluU2l6ZSwKICBwbG90LnJhbmdlID0gYygzLDExLDMsMTEpCikKCiMjIyBQTE9UIEhFQVRNQVAKZmNzJGJpbnBsb3RfZnJlcV9kb3VibGVwb3MoCiAgZGF0YSA9IGV4cHJzLnF1YWQsCiAgZmVhdC5YID0gZmVhdFgsCiAgZmVhdC5ZID0gZmVhdFksCiAgZmVhdC5aMSA9IGZlYXRaMSwKICBmZWF0LloyID0gZmVhdFoyLAogIGN1dG9mZnMgPSBjdXRvZmZzLm1hbiwKICBiaW5zaXplID0gYmluU2l6ZSwKICBtYXhmcmVxID0gbWF4ZnJlcSwKICBwbG90LnJhbmdlID0gYygzLDExLDMsMTEpCikKYGBgCgpJbiByZWdhcmRzIHRvIDNELXN1cmZhY2UgcGxvdHMsIGZyZXF1ZW5jeSBiaW4gdGFibGVzIGFyZSBwcm92aWRlZCBhbmQgZXhwb3J0ZWQgZnJvbSBQUkkgYW5kIGNyZWF0ZWQgd2l0aCBwYWNrYWdlICdwbG90bHknIHY0LjcuMS4gRXhhbXBsZXMgYXJlIHNob3duIGZyb20gRmlndXJlIDZnLgoKYGBge3Igc3VyZmFjZSBwbG90c30KbGlicmFyeShwbG90bHkpCmZlYXRYID0gIlBEMSIKZmVhdFkgPSAiSUZOZyIKZmVhdFoxID0gIkJjbDYiCmN1dG9mZnMubWFuID0gYyg2Ljk4Miw3LjQ3OSw3LjM0NikKYmluU2l6ZSA9IDAuMgoKYmlucGxvdC50YWJsZS5aMSA9IGZjcyRiaW5wbG90X3RhYmxlKAogIGRhdGEgPSBleHBycy5xdWFkLCAKICBmZWF0LlggPSBmZWF0WCwgCiAgZmVhdC5ZID0gZmVhdFksIAogIGZlYXQuWjEgPSBmZWF0WjEsIAogIGNhbGMgPSAiZnJlcSIsCiAgYmluc2l6ZSA9IGJpblNpemUsCiAgY3V0b2ZmcyA9IGN1dG9mZnMubWFuLAogIHBsb3R0aW5nID0gRkFMU0UKKQoKZmVhdFoyID0gIklMMjEiCmN1dG9mZnMubWFuID0gYyg2Ljk4Miw3LjQ3OSw3LjY5NikKCmJpbnBsb3QudGFibGUuWjIgPSBmY3MkYmlucGxvdF90YWJsZSgKICBkYXRhID0gZXhwcnMucXVhZCwgCiAgZmVhdC5YID0gZmVhdFgsIAogIGZlYXQuWSA9IGZlYXRZLCAKICBmZWF0LloxID0gZmVhdFoyLCAKICBjYWxjID0gImZyZXEiLAogIGJpbnNpemUgPSBiaW5TaXplLAogIGN1dG9mZnMgPSBjdXRvZmZzLm1hbiwKICBwbG90dGluZyA9IEZBTFNFCikKCgp4PXNlcSgyLDEyLGJ5PTAuMikKeT1zZXEoMiwxMixieT0wLjIpCnNjZW5lID0gbGlzdCh4YXhpcz1saXN0KHRpdGxlPSAiUEQtMSIpLAogICAgeWF4aXM9bGlzdCh0aXRsZT0iSUZOLWciKSwKICAgIHpheGlzPWxpc3QodGl0bGU9ImZyZXEiLHJhbmdlPWMoMCw4NSkpLAogICAgY2FtZXJhcG9zaXRpb249bGlzdChjKC0wLjEsIDAuNCwgLTEsIDAuMzMpLCBjKDAuMCwgMCwgMC4wKSwgMikKKQojIyMgVU5DT01NRU5UIFRPIFBMT1QKIyMjIEZpZ3VyZSB3aXRoIEJjbDYKIyBwbG90X2x5KHNob3dzY2FsZSA9IFRSVUUpICU+JSBhZGRfc3VyZmFjZSh4PXgseT15LHo9dChiaW5wbG90LnRhYmxlLloxKSwgY21pbj0wLCBjbWF4PTgyLGNvbG9ycz0iR3JlZW5zIixyZXZlcnNlc2NhbGU9RikgJT4lIGxheW91dChzY2VuZT1zY2VuZSkKIyMjIEZpZ3VyZSB3aXRoIElMMjEgCiMgcGxvdF9seShzaG93c2NhbGUgPSBUUlVFKSAlPiUgYWRkX3N1cmZhY2UoeD14LHk9eSx6PXQoYmlucGxvdC50YWJsZS5aMiksIGNtaW49MCwgY21heD04Mixjb2xvcnM9IkdyZWVucyIscmV2ZXJzZXNjYWxlPUYpICU+JSBsYXlvdXQoc2NlbmU9c2NlbmUpCmBgYAo=
