# Supplementary material for: Identification of a super-functional Tfh-like subpopulation in murine lupus by pattern perception
Source: eLife. 2020 May 22;9:e53226. doi: 10.7554/eLife.53226 (PMC7274784; doi:10.7554/eLife.53226)
Supplement: Supplementary file 1. [file elife-53226-supp1.docx]

| **Key Resources Table** | | | | |
| --- | --- | --- | --- | --- |
| **Reagent type (species) or resource** | **Designation** | **Source or reference** | **Identifiers** | **Additional information** |
| antibody | APC conjugated Recombinant monoclonal anti-Bcl6 (clone REA373) | Miltenyi | Cat#130-105-485; RRID: AB_2651272 | (1:15) |
| antibody | PE conjugated Hamster monoclonal anti-BTLA (CD272, clone HMBT-6B2) | BD Biosciences | Cat#563774; RRID AB_2738420 | (1:100) |
| antibody | FITC conjugated rat monoclonal anti-mouse TNF-α (clone MP6-XT22) | DRFZ | DRFZ_ID#2308 | (0.65 μg/ml)  Produced and purified from hybridoma, flurochome-conjugated according to standard procedures |
| antibody | Rat monoclonal anti-CD3 (clone KT3) | DRFZ | DRFZ_ID#1916 | (2 μg/ml) Produced and purified from hybridoma, flurochome-conjugated according to standard procedures |
| antibody | Alexa700-conjugated  Rat monoclonal anti-CD3 (clone KT3) | DRFZ | DRFZ_ID#2861 | (0.58 μg/ml) Produced and purified from hybridoma, flurochome-conjugated according to standard procedures |
| antibody | PerCP-Cy5.5 conjugated Rat monoclonal anti-CD4 (clone RM4-5) | eBioscience | Cat#45-0042-82  RRID AB_1107001 | (1:200) |
| antibody | FITC conjugated Rat monoclonal anti-CD4 (clone YTS19.1) | DRFZ | DRFZ_ID#130317 | (2.15 μg/ml) Produced and purified from hybridoma, flurochome-conjugated according to standard procedures |
| antibody | Pac Blue conjugated Rat monoclonal anti-CD4 (clone GK1.5) | DRFZ | DRFZ_ID#2723 | (0.5 μg/ml) Produced and purified from hybridoma, flurochome-conjugated according to standard procedures |
| antibody | PE conjugated Rat monoclonal anti-CD4 (clone GK1.5) | DRFZ | DRFZ_ID#2848 | (0.86 μg/ml) Produced and purified from hybridoma, flurochome-conjugated according to standard procedures |
| antibody | FITC conjugated Rat monoclonal anti-B220 (clone RA3.6B2) | DRFZ | DRFZ_ID#2826 | (2.12 μg/ml) Produced and purified from hybridoma, flurochome-conjugated according to standard procedures |
| antibody | APC conjugated Hamster monoclonal anti-CD27 (clone LG.3A10) | Miltenyi | Cat#130-100-441; RRID: AB_2656821 | (1:80) |
| antibody | Pac Blue conjugated Rat monoclonal anti-CD44 (clone IM7) | DRFZ | DRFZ_ID#2779 | (2.24 μg/ml) Produced and purified from hybridoma, flurochome-conjugated according to standard procedures |
| antibody | BV785 conjugated Rat monoclonal anti-CD44 (clone IM7) | Biolegend | Cat#103059; RRID: AB_2571953 | (1:400) |
| antibody | APC conjugated Hamster monoclonal anti-CD40L (CD154, clone MR1) | eBioscience | Cat#17-1541-82; RRID: AB_795823 | (1:100) |
| antibody | APC conjugated Hamster monoclonal anti-CTLA4 (clone UC10-4F10-11) | BD Biosciences | Cat#564331; RRID: AB_2738751 | (1:100) |
| antibody | APC conjugated Hamster monoclonal anti-CXCR3 (clone CXCR3-173) | eBioscience | Cat#17-1831-80; RRID: AB_1210792 | (1:150) |
| antibody | BV421 conjugated Hamster monoclonal anti-mouse CD183 CXCR3 antibody (clone  CXCR3-173) | BioLegend | Cat# 126522; RRID:AB_2562205 | (1:100) |
| antibody | APC conjugated Hamster monoclonal anti-mouse CD183 (CXCR3) antibody  (clone CXCR3-173) | BioLegend | Cat# 126512; RRID:AB_1088993 | (1:200) |
| antibody | APC conjugated Rat monoclonal anti-CXCR4 (clone L276F12) | BioLegend | Cat#146507; RRID: AB_2562784 | (1:100) |
| antibody | BV605 conjugated Rat monoclonal anti-CXCR5 (CD185, clone L138D7) | Biolegend | Cat#145513; RRID: AB_2562208 | (1:320) |
| antibody | Rat monoclonal anti-CXCR5 (CD185, clone L138D7) | BioLegend | Cat# 145517; RRID:AB_2562453 | (1:300) |
| antibody | APC conjugated Rat monoclonal anti-FoxP3 (clone FJK-16s) | eBioscience | Cat#17-5773-82; RRID: AB_469457 | (1:100) |
| antibody | FITC conjugated Rat monoclonal anti-FoxP3 (clone FJK-16s) | eBioscience | Cat#11-5773-82,; RRID: AB_465243 | (1:100) |
| antibody | PE conjugated Rat monoclonal anti-GITR (clone DTA-1) | eBioscience | Cat#12-5874-82; RRID: AB_465986 | (1:500) |
| antibody | PE-Cy7 conjugated Hamster monoclonal anti-ICOS (clone C398.4A) | eBioscience | Cat#25-9949-82; RRID: AB_2573566 | (1:50) |
| antibody | PE-Cy7 conjugated Rat monoclonal anti-IFN-γ (clone XMG1.2) | BD Biosciences | Cat#557649; RRID: AB_396766 | (1:400) |
| antibody | FITC conjugated Rat monoclonal anti-IFN-γ (clone AN18.17.24) | DRFZ | DRFZ_ID#2349 | (0.425 μg/ml) Produced and purified from hybridoma, flurochome-conjugated according to standard procedures |
| antibody | Rat monoclonal anti-IFN-γ (clone AN18.17.24) | DRFZ | DRFZ_ID#2846 | (10 μg/ml) Produced and purified from hybridoma, flurochome-conjugated according to standard procedures |
| antibody | Rat monoclonal anti-CD40 (clone FGK-45) | DRFZ | DRFZ_ID#1345 | (10 μg/ml) Produced and purified from hybridoma, flurochome-conjugated according to standard procedures |
| antibody | BV510 conjugated Rat monoclonal anti-IL-2 (clone JES6-5H4) | Biolegend | Cat#503833; RRID: AB_2562977 | (1:400) |
| antibody | APC conjugated Rat monoclonal anti-IL-2 (clone JES6-5H4) | eBioscience | Cat#17-7021-82; RRID: AB_469490 | (1:100) |
| antibody | APC conjugated Rat monoclonal anti-IL-10 (clone JES5-16E3) | eBioscience | Cat#17-7101-82; RRID: AB_469502 | (1:100) |
| antibody | PerCP-Cy5.5 conjugated Rat monoclonal anti-IL-10 (clone JES5-16E3) | BioLegend | Cat#505028; RRID: AB_2561523 | (1:100) |
| antibody | PE conjugated Rat monoclonal anti-IL21 (clone mhalx21) | Thermo | Cat#12-7213-82; RRID: AB_1834465 | (1:50-1:200) |
| antibody | FITC conjugated Rat monoclonal anti-Lag3 (clone C9B7W) | eBioscience | Cat#11-2231-82; RRID: AB_2572484 | (1:100) |
| antibody | PE-Cy7 conjugated Rat monoclonal anti-OX40 (CD134, clone OX-86) | BioLegend | Cat#119416; RRID: AB_2566155 | (1:200) |
| antibody | PerCP-Cy5.5. conjugated Hamster monoclonal anti-PD-1 (CD279, clone J43) | eBioscience | Cat#46-9985-82; RRID: AB_11150055 | (1:200) |
| antibody | PE conjugated Hamster monoclonal anti-PD-1 (CD279, clone J43) | eBioscience | Cat#12-9985-82; RRID: AB_466295 | (1:200) |
| antibody | APC-Cy7 conjugated anti-mouse CD279 (PD-1) antibody (clone 29F.1A12) | BioLegend | Cat# 135223, RRID:AB_2563522 | (1:200) |
| antibody | PerCP-Cy5.5 conjugated Rat monoclonal anti-PSGL1 (clone 2PH1) | BD Biosciences | Cat#564310; RRID: AB_2738736 | (1:400) |
| antibody | PE conjugated Mouse monoclonal anti-Tbet (clone 4B10) | BioLegend | Cat#644810; RRID: AB_2200542 | 1:20 |
| antibody | PE conjugated Mouse monoclonal anti-Tbet (clone 4B10) | eBioscience | Cat#12-5825-82; RRID: AB_925761 | (1:100) |
| antibody | PE conjugated Mouse monoclonal anti-TIGIT (clone 1G9) | BioLegend | Cat#142103; RRID: AB_10895760 | (1:50) |
| antibody | FITC conjugated Rat monoclonal anti-TNF-α (clone MP6-XT22) | DRFZ | DRFZ_ID#2308 | (0.65 μg/ml) Produced and purified from hybridoma, flurochome-conjugated according to standard procedures |
| antibody | Goat polyclonal anti-IgG conjugated to Biotin | Southern Biotech | Cat#1030-08; RRID: AB_2103446 | 1:2000 |
| antibody | Goat polyclonal anti-IgM conjugated to Biotin | Southern Biotech | Cat#1020-08; RRID: AB_616726 | 1:2000 |
| antibody | Hamster monoclonal anti-FAS (clone Jo2) | BD Biosciences | Cat# 557653; RRID: AB_396768 | (1:200) |
| antibody | Rat anti-mouse/human GL7 Antigen | BioLegend | Cat# 144610; RRID:AB_2562979 | (1:300) |
| antibody | Hamster anti-mouse CD28 Antigen | BioLegend | Cat# 102116; RRID: AB_11147170 | (5 μg/ml) |
| chemical compound, drug | Pacific Orange™ Succinimidyl Ester, Triethylammonium Salt | Thermo Scientific | Cat#30253 | Staining of dead cells  (1µg/ml) |
| chemical compound, drug | Ionomycin | Merck | Cat#407952; CAS: 56092-82-1 | T cell stimulator |
| chemical compound, drug | Brefeldin A | Sigma-Aldrich | Cat#B7651; CAS: 20350-15-6 | Golgi inhibitor |
| chemical compound, drug | PMA | Sigma-Aldrich | Cat#P8139; CAS: 16561-29-8 | T cell stimulator |
| chemical compound, drug | methylated BSA | SIGMA | Cat#A1009-250mg |  |
| chemical compound, drug | dsDNA | SIGMA | Cat#D4522-1mg; CAS: 91080-16-9 |  |
| chemical compound, drug | ExtrAvidin-Peroxidase | SIGMA | Cat#E2886 |  |
| chemical compound, drug | 1-step ultra TMB-ELISA | Thermo Scientific | Cat#34028 |  |
| chemical compound, drug | 4′,6-diamidino-2-phenylindole (DAPI) | ThermoFisher Scientific | D1306 | Staining of dead cells |
| chemical compound, drug | Propidium Iodide | SIGMA | P4864-10ML | Staining of dead cells |
| chemical compound, drug | L/D aqua fixable dye | ThermoFisher Scientific | Cat#L34957 | Staining of dead cells |
| commercial assay or kit | Uristix | Siemens | Cat#2857 |  |
| commercial assay or kit | Foxp3 / Transcription Factor Staining Buffer Set | eBioscience | Cat#00-5523-00 |  |
| commercial assay or kit | Mouse IgG ELISA Kit | LifeSpan Biosciences | LS-F10451-1 |  |
| Deposited Data | Flow cytometry data | This study | Planned to be deposited on FlowRepository |  |
| strain, strain background (Mus musculus) | Mouse/NZBWF1/J | Hybrid cross between NZB/BlNJ (000684) Female x NZW/LacJ (001058) Male;  BfR Breeding colony |  | mouse |
| strain, strain background (Mus musculus) | Mouse/C57BL/6JRj | Janvier Labs (France) |  | mouse |
| Software and Algorithms | FlowJo v10.0.7 | FlowJo LLC | www.flowjo.com |  |
| Software and Algorithms | flowCore v1.36.9 (R package) | Ellis et al., 2019 | https://www.bioconductor.org/packages/release/bioc/html/flowCore.html |  |
| Software and Algorithms | R 3.2.4 Revised | R Development Core Team, 2015 | R-project.org |  |
| Software and Algorithms | Prism Version 7 | Graphpad | https://www.graphpad.com/scientific-software/prism/ |  |
| Software and Algorithms | PRI | This study |  | Written in R, see source code files |
